# Subanesthetic isoflurane relieves zymosan-induced neutrophil inflammatory response by targeting NMDA glutamate receptor and Toll-like receptor 2 signaling

**DOI:** 10.18632/oncotarget.9091

**Published:** 2016-04-28

**Authors:** Jun-Tang Li, Wei-Qi Wang, Ling Wang, Ning-Ning Liu, Ya-Li Zhao, Xiao-Shan Zhu, Qin-Qin Liu, Chun-Fang Gao, An-Gang Yang, Lin-Tao Jia

**Affiliations:** ^1^ Centre of Inflammation and Cancer Research, 150th Central Hospital of PLA, Luoyang, Henan, China; ^2^ State Key Laboratory of Cancer Biology, Department of Immunology, Fourth Military Medical University, Xi'an, Shaanxi, China; ^3^ State Key Laboratory of Cancer Biology, Department of Biochemistry and Molecular Biology, Fourth Military Medical University, Xi'an, Shaanxi, China; ^4^ State Key Laboratory of Military Stomatology, Department of Oral and Maxillofacial Surgery, School of Stomatology, Fourth Military Medical University, Xi'an, China; ^5^ National Clinical Research Center for Oral Diseases, Department of Oral and Maxillofacial Surgery, School of Stomatology, Fourth Military Medical University, Xi'an, China; ^6^ Shaanxi Clinical Research Center for Oral Diseases, Department of Oral and Maxillofacial Surgery, School of Stomatology, Fourth Military Medical University, Xi'an, China; ^7^ Department of Anesthesiology, 150th Central Hospital of PLA, Luoyang, Henan, China

**Keywords:** isoflurane, zymosan, neutrophil, NMDA receptor, TLR2, Immunology and Microbiology Section, Immune response, Immunity

## Abstract

Neutrophil release of NO/ONOO^−^ induces endothelial cell barrier dysfunction in inflammatory acute lung injury (ALI). Previous studies using zymosan-triggered inflammation and ALI model revealed that zymosan promotes inducible NO synthase (iNOS) expression in neutrophils, and that isoflurane inhibits zymosan-induced oxidative stress and iNOS biosynthesis. However, the underlying mechanisms remain largely unknown. We found here that in zymosan-primed neutrophils, iNOS is transcriptionally activated by NF-κB, whose nuclear translocation is triggered by excessive reactive oxygen species (ROS) and consequently activated p38 MAPK. ROS production is attributed to zymosan-initiated Toll-like receptor 2 (TLR2) signaling, in which the adaptor MyD88 recruits and activates c-Src, and c-Src activates NADPH oxidase to generate ROS. Subanesthetic isoflurane counteracts the aforementioned zymosan-induced signaling by targeting N-methyl-D-aspartic acid (NMDA) glutamate receptor and thereby suppressing calcium influx and c-Src activation. Whereas iNOS accelerates NO/ONOO^−^ production in neutrophils which eventually promote protein leak from pulmonary microvascular endothelial cells (PMVEC), isoflurane reduced NO/ONOO^−^ release from zymosan-treated neutrophils, and thus relieves trans- PMVEC protein leak. This study provides novel insights into the roles of neutrophils and the underlying mechanisms in zymosan-induced ALI, and has implications for the therapeutic potential of subanesthetic isoflurane in attenuating inflammatory responses causing lung endothelial cell damage.

## INTRODUCTION

Acute lung injury (ALI) is a severe disorder that causes profound morbidity and 30-40% mortality, and exhibits high incidence of progressing into acute respiratory distress syndrome (ARDS) [1]. Neutrophils play a crucial role in the ALI inflammatory response, which is predominantly mediated through iNOS induction and nitric oxide (NO)/peroxynitrite (ONOO^−^) generation [2, 3]. Zymosan is a substance derived from the cell walls of the yeast *Saccharomyces cerevisiae* [4, 5]. Zymosan-induced ALI, in which neutrophils infiltrate into the lungs and contribute to the oxidation-induced lung damage, has emerged as a classical model for inflammation-related tissue injury [5]. Zymosan-triggered inflammatory cascade leads to high-permeability pulmonary edema, largely through activation and injury of endothelial cells, specifically the pulmonary microvascular endothelial cells (PMVECs) [6]. We and others have demonstrated that NO/ONOO^−^ release from neutrophils underlies various pathophysiological events involved in ALI including pulmonary neutrophil infiltration, oxidant stress, and microvascular protein leak [7, 8]. NF-κB was reported to transcriptionally activate iNOS in several NO-producing cell types [9]; Nicotinamide adenine dinucleotidephosphate (NADPH) oxidase-derived ROS induce iNOS expression in neutrophils, leading to lung fibrosis [10]. Nevertheless, how these signaling events are connected to switch on iNOS expression remains elusive.

Isoflurane is a widely used inhaled anesthetic, which reduces pain sensitivity *via* modulation of the neurotransmitter receptors, and exerts protective properties through antioxidant and anti-inflammatory effects [11, 12]. Prospects for the clinical usage of isoflurane (1.2-2.5%) have been hampered due to its adverse systemic effects [11]; however, our previous studies have shown that subanesthetic isoflurane (0.7%) protects against zymosan-induced lung injury by upregulating antioxidant enzymes and inhibiting inflammatory responses *via* the reduction of iNOS induction and NO production in neutrophils [7]. Moreover, subanesthetic isoflurane reduces zymosan- induced inflammation in Kupffer cells by inhibiting the NF-κB pathway [13]. In addition, isoflurane preserves ATP-sensitive K^+^ channel activity in the human omental artery during oxidative stress induced by high glucose, which is mediated by NADPH oxidase inhibition [14]. However, it is unclear how subanesthetic isoflurane affects the NADPH oxidase and NF-κB activities, and whether these mechanisms are involved in isoflurane alleviation of zymosan-induced iNOS expression and NO/ONOO^−^ release in neutrophils which mediate endothelial damage.

In this study, we found that zymosan initiates Toll-like receptor 2 (TLR2) signaling in neutrophils, which recruits and activates c-Src *via* the adaptor MyD88; c-Src triggers cytomembrane localization of the NADPH oxidase subunit, p47^phox^, leading to excessive ROS production and p38 MAPK activation [15]. p38 MAPK subsequently activates NF-κB to switch on iNOS expression, which promotes the synthesis and release of NO/ONOO^−^ in neutrophils, and eventually causes the transmembrane protein leak from pulmonary microvascular endothelial cells (PMVEC). Subanesthetic isoflurane protects against trans-PMVEC protein leak by targeting the N-methyl-D-aspartic acid (NMDA) glutamate receptor and thereby suppressing calcium signaling and c-Src activation in neutrophils.

## RESULTS

### Opposite roles of zymosan and subanesthetic isoflurane in neutrophil-mediated trans-PMVEC protein leak

**Figure 1:**
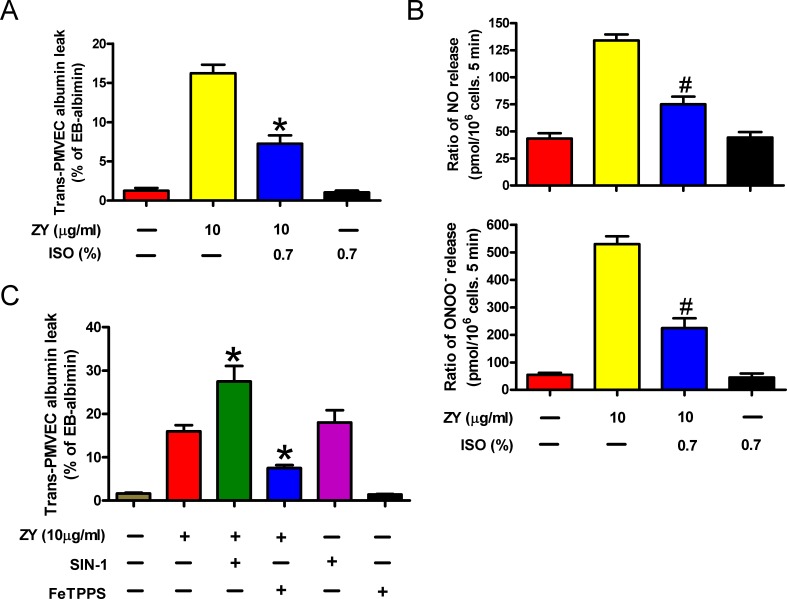
The capacity of zymosan-stimulated neutrophils to induce trans-PMVEC protein leak is inhibited by subanesthetic isoflurane **A.** Neutrophils were sequentially treated with zymosan (ZY) for 15 min and 0.7% isoflurane (ISO) for 15 min where indicated, followed by incubation with zymosan for 12 h. Treatment of cells with solvent serves as control groups for zymosan and isoflurane. The media were collected and added to the monolayer PMVEC. Trans-PMVEC EB-albumin leak was determined. **B.** Neutrophils were treated as described in (A), followed by measurement of NO production and ONOO^−^ release by neutrophils. **C.** Neutrophils were treated with zymosan or solvent for 12 h, followed by treatment with or without FeTPPS (25 mM) or SIN-1 (200 mM) for 15 min. The media were replaced with fresh complete media, which were collected 6 h later and added to the monolayer PMVEC. Trans-PMVEC EB-albumin leak was determined. Data are represented as the mean ± SEM of 3 replicates. *P < 0.05, ^#^*P* < 0.01, as compared with zymosan group.

ALI is characterized by PMVEC injury leading to high protein pulmonary edema. It has been reported that trans- PMVEC albumin leak under septic conditions is dependent on iNOS activity specifically in neutrophils, but not in PMVECs themselves. Septic neutrophil-dependent trans-PMVEC albumin leak may be mediated by peroxynitrite [16]. Consistently, we found here that zymosan treatment had no effect on iNOS protein expression and NO and ONOO^−^ production in PMVECs, but significantly enhanced the levels of iNOS protein and NO and ONOO^−^ in neutrophils (Supplementary Figure S1). Neutrophils are critically involved in zymosan-triggered inflammatory responses leading to endothelial protein leak and ALI, whereas subanesthetic isoflurane relieves zymosan-induced endothelial damage [5, 17]. To investigate the effect of neutrophil priming on trans-PMVEC Evans Blue (EB)-albumin leak, we cultured mouse PMVECs with the conditioning media of zymosan- and/or isoflurane-treated neutrophils. As a result, the media of zymosan-stimulated neutrophils elicited remarkable albumin leak from the PMVECs (Figure 1A). Compared with zymosan treatment alone, combined treatment of neutrophils with zymosan and subanesthetic isoflurane (0.7%) produced conditioned media which caused decreased albumin leak by PMVECs (Figure 1A). In zymosan-induced ALI, neutrophils were reported to induce vascular leak by releasing peroxynitrite [18]. Indeed, we detected dramatically increased NO and ONOO^−^ production by neutrophils upon stimulation with zymosan, whereas combined treatment of neutrophils with isoflurane reduced NO and ONOO^−^ levels in neutrophils (Figure 1B). The albumin leak by PMVECs was further enhanced by incorporating in the media an ONOO^−^ donor, SIN-1, and was inhibited by an ONOO^−^ scavenger, FeTPPS (Figure 1C). These data suggest that zymosan-primed neutrophils cause trans-PMVEC protein leak by producing NO and ONOO^−^, a process suppressed by isoflurane treatment of neutrophils.

### Isoflurane counteracts zymosan-induced NF-κB transactivation of iNOS

**Figure 2:**
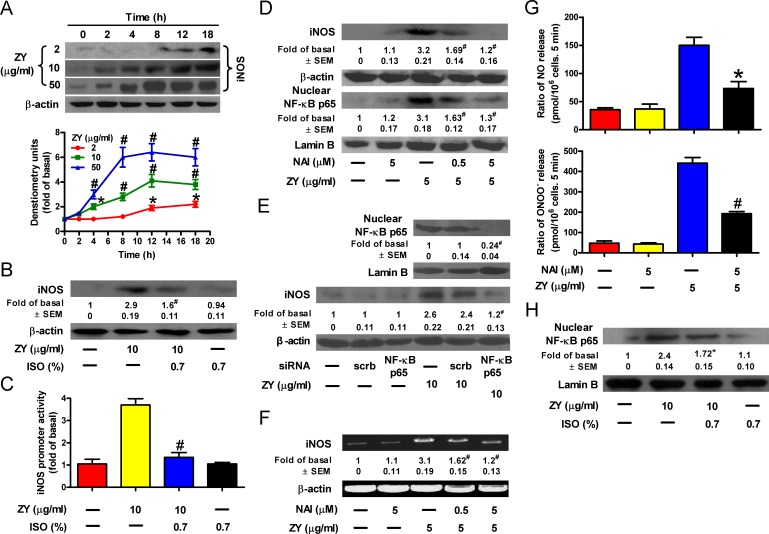
Isoflurane impairs zymosan-induced NF-κB activation, iNOS expression and NO/ONOO^−^ production in neutrophils **A.** Neutrophils were treated with increasing concentrations of zymosan for indicated time periods, and were subjected to Western blot analysis. **B.** Neutrophils were incubated with zymosan for 15 min, post-treated with 0.7% isoflurane for 15 min, followed by a continuous incubation with zymosan for a total of 8 h before Western blot analysis. β-actin was used as an inner control for whole cell lysates. **C.** Neutrophils were co-transfected with or without an iNOS-luc reporter plasmid and indicated siRNAs. The promoter activity of iNOS was determined in the cell lysates after zymosan treatment for 6 h. **D.**, **E.** Neutrophils were pretreated with NAI (D) or transfected with NF-κB p65 siRNA (E), followed by incubation with zymosan for 8 h. The nuclear or whole cell lysates were prepared for Western blot analysis. Lamin B was used as an inner control for the lysates of the nuclear fraction. **F.** Neutrophils were treated as described in (D), followed by incubation with zymosan for 6 h prior to RT-PCR analysis. **G.** Neutrophils were treated as described in (D), followed by measurement of NO and ONOO^−^ production. **H.** Neutrophils were treated as described in (B), and the nuclear lysates were prepared for Western blot analysis. Data are represented as the mean ± SEM of 3 replicates or representative of 3 independent experiments. *P < 0.05, ^#^*P* < 0.01, as compared with the cells exposed to vehicle alone (A). *P < 0.05, ^#^*P* < 0.01, as compared with the cells exposed to zymosan alone (B-H), or zymosan + scrambled siRNA (E).

Zymosan was reported to promote NO production by upregulating iNOS [19]. Consistently, we observed that zymosan induced iNOS expression in a time- and dose-dependent manner in neutrophils (Figure 2A). However, when these neutrophils were further treated with isoflurane, zymosan-induced iNOS expression in neutrophils was significantly reduced (Figure 2B). These results are in agreement with the modulation of iNOS promoter activity by zymosan and/or isoflurane in neutrophils (Figure 2C). iNOS is a known target gene of NF-κB, the key transcriptional factor involved in inflammatory signaling [20]. Thus, we investigated whether NF-κB plays a role in zymosan-induced iNOS expression in neutrophils. We found that zymosan- induced iNOS expression is accompanied by an increase in the nuclear levels of the NF-κB subunit, p65 (Figure 2D). In addition, iNOS expression was hindered by p65 knockdown and by the NF-κB inhibitor, NAI, in a concentration-dependent manner (Figures 2D and 2E). The alterations in iNOS mRNA levels are consistent with those of the iNOS protein, suggesting that NF-κB transcriptionally activates iNOS in zymosan-treated neutrophils (Figure 2F). Subsequently, treatment of neutrophils with NAI significantly reduced zymosan-induced NO and ONOO^−^ release (Figure 2G). Consistent with its inhibitory role in iNOS expression and NO and ONOO^−^ production by neutrophils, subanesthetic isoflurane treatment significantly impeded zymosan-induced nuclear translocation of p65 (Figure 2H). Therefore, isoflurane and zymosan inversely regulate iNOS expression by targeting NF-κB in neutrophils.

### ROS/p38 MAPK signaling is required for isoflurane and zymosan regulation of NF-κB

**Figure 3:**
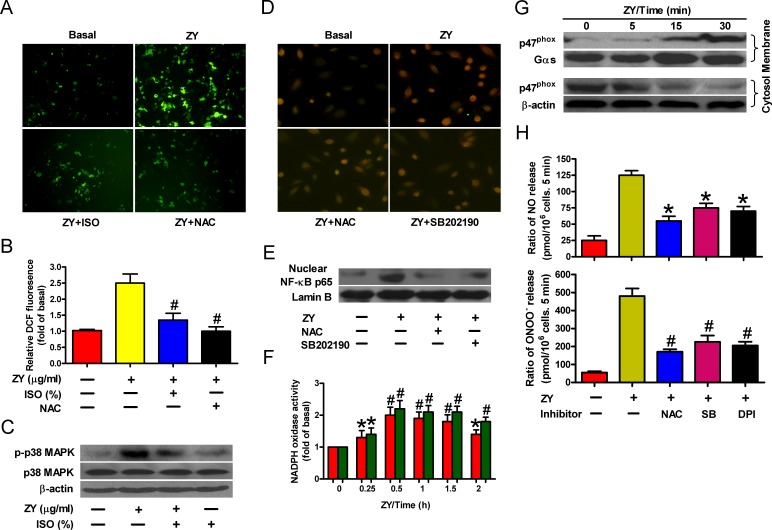
Isoflurane and zymosan regulate NF-κB activation *via* ROS/p38 MAPK signaling **A.**, **B.** Neutrophils were sequentially treated with zymosan (10 μg/ml) for 15 min, 0.7% isoflurane for 15 min where indicated, and zymosan for 6 h in the presence or absence of NAC (50 mM). Cells were subjected to fluorescence measurement of H_2_O_2_ levels (A) and Western blot analysis (B). Scale bar = 10 μM. **C.**, **D.** Neutrophils were treated with zymosan for 6 h in the presence of the p38 MAPK inhibitor SB202190 (10 mM) where indicated, followed by immunofluorescent staining of p65 (C) and Western blot analysis of nuclear p65 levels (D). Scale bar = 10 μM. **E.**, **F.** Neutrophils were treated with zymosan for indicated time periods, followed by measurement of NADPH oxidase activity (E) or Western blot assay using the membrane and cytosol fractions of cell lysates (F). Gas and β-actin were used as inner controls for membrane and cytosolic fractions, respectively. **G.** Neutrophils were treated with zymosan for 12 h in the presense of NAC (50 mM), SB202190 (10 mM) or NADPH oxidase inhibitor DPI (10 mM) where indicated, followed by measurement of NO and ONOO^−^ production. Data are represented as the mean ± SEM of 3 replicates or representative of 3 independent experiments. *P < 0.05, ^#^*P* < 0.01, as compared with the cells exposed to zymosan alone (B, C, E, H). *P < 0.05, ^#^*P* < 0.01, as compared with the cells exposed to vehicle alone (F, G).

NF-κB can be activated by inflammatory mediators *via* various signal pathways [21]. In particular, ROS-activated p38 MAPK triggers the nuclear translocation of NF-κB by phosphorylating and degrading its inhibitory partner, IκB [21]. To test whether this is involved in zymosan-induced NF-κB activation, we measured the intracellular ROS levels in neutrophils in response to zymosan stimulation. As shown in Figure 3A, zymosan significantly enhanced ROS levels in neutrophils, which can be dampened by treatment with isoflurane or the ROS scavenger NAC. Furthermore, zymosan induced the phosphorylation of p38 MAPK, whereas isoflurane impaired p38 MAPK activation probably by suppressing ROS production (Figure 3B). ROS production and p38 MAPK activation are required for NF-κB translocation because NAC or the p38 MAPK inhibitor SB202190 efficiently abrogated zymosan-elicited nuclear enrichment of NF-κB in neutrophils (Figures 3C and 3D). Thus, zymosan evokes and isoflurane impedes NF-κB activation by dictating ROS levels in neutrophils.

NADPH oxidase plays a crucial role in intracellular ROS production by coupling transmembrane transfer of NADPH electrons and generation of superoxide anions which can be converted to hydrogen peroxide and other forms of ROS [22]. To this end, we observed a substantial elevation of NADPH oxidase activity in zymosan-treated neutrophils (Figure 3E). In addition, zymosan induced the membrane translocation of the cytosolic subunit p47^phox^, which is required for NADPH oxidase activation (Figure 3F) [23]. Consistent with these observations, we found that ROS scavenging or p38 MAPK inhibition, or treatment of cells with the NADPH oxidase inhibitor DPI, significantly reduced the production of NO and ONOO^−^ by zymosan-stimulated neutrophils (Figure 3G). These data suggest that NADPH oxidase/ROS/p38 MAPK signaling is responsible for NF-κB activation and NO/ONOO^−^ release of zymosan-primed neutrophils.

### c-Src mediates ROS production in isoflurane and zymosan-treated neutrophils

**Figure 4:**
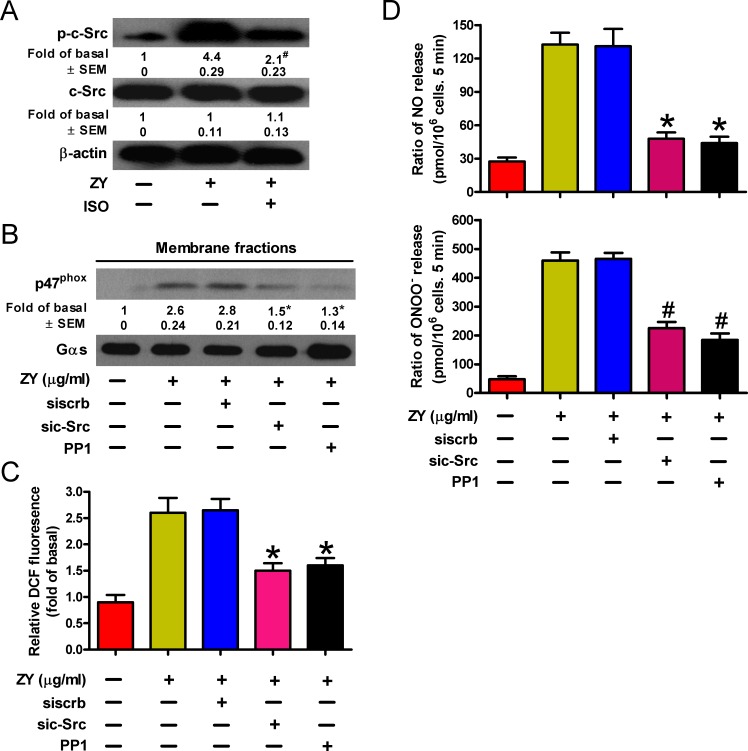
ROS production is dependent on c-Src activity inversely regulated by isoflurane and zymosan in neutrophils **A.** Neutrophils were sequentially treated with zymosan (10 μg/ml) for 15 min, 0.7% isoflurane for 15 min where indicated, and zymosan for another 6 h prior to Western blot analysis. **B.**-**D.** Neutrophils were transfected with c-Src-targeted (sic-Src) or scrambled (siscrb) siRNAs, and treated with zymosan and/or the Src inhibitor PP1 (5 mM) for 6 h. Cells were then subjected to Western blot analysis of the membrane fraction lysates (B), fluorescent assay of H_2_O_2_ levels (C), and measurement of NO and ONOO^−^ production (D). Data are represented as the mean ± SEM of 3 replicates or representative of 3 independent experiments. *P < 0.05, ^#^*P* < 0.01, as compared with the cells exposed to zymosan alone (A-D), or zymosan + scrambled siRNA (B-D).

NADPH oxidase is activated by various stress stimuli *via* the protein kinase c-Src [24]. We next investigated whether c-Src activation is involved in NADPH oxidase activation and ROS production of zymosan- and/or isoflurane-treated neutrophils. As shown in Figure 4A, zymosan induced the phosphorylation of c-Src in neutrophils, which was inhibited by incubation of cells with subanesthetic isoflurane. c-Src activity is required for zymosan-induced signaling for peroxynitrite release by neutrophils given that both siRNAs and inhibitors of c-Src can ablate the membrane translocation of p47^phox^ (Figure 4B), the production of ROS (Figure 4C), and eventually the cellular levels of NO and ONOO^−^ (Figure 4D). Thus, c-Src may serve as a convergent target of zymosan and isoflurane in regulating the neutrophil release of endothelium-damaging metabolites.

### Zymosan activates c-Src in neutrophils *via* TLR2 signaling

**Figure 5:**
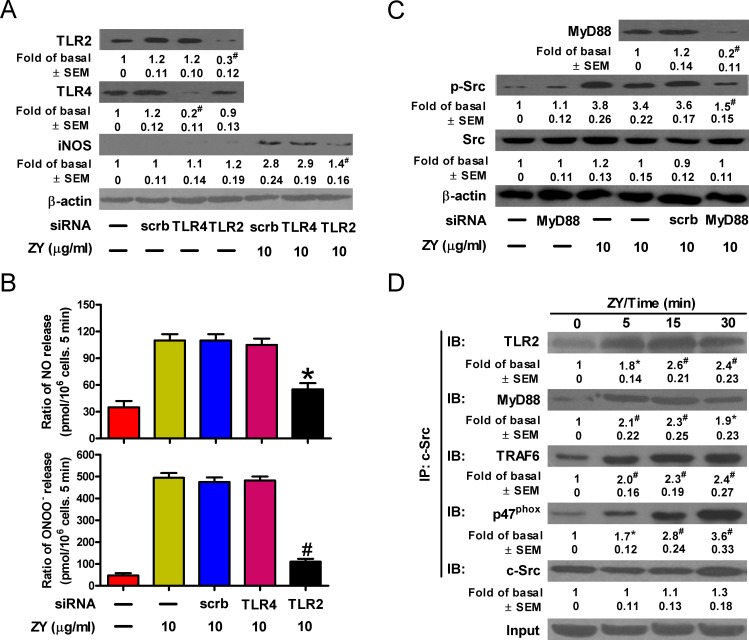
Zymosan activates c-Src through TLR2 signaling in neutrophils **A.**-**C.** Neutrophils were transfected with indicated siRNAs prior to treatment with or without zymosan for 6 h. Cells were then subjected to Western blot analysis (A, C) and measurement of NO and ONOO^−^ production (B). **D.** Neutrophils were treated with zymosan (10 μg/ml) for indicated time periods. Cell lysates were prepared and immunoprecipitated using c-Src antibody, followed by Western blot analysis of the precipitated proteins. Data are represented as the mean ± SEM of 3 replicates or representative of 3 independent experiments. ^#^*P* < 0.01, as compared with the cells exposed to scrambled siRNA alone [A (upper panel)]. *P < 0.05, ^#^*P* < 0.01, as compared with the cells exposed to zymosan alone (B, C), zymosan + scrambled siRNA [A (lower panel), B, C], or vehicle alone (D).

Zymosan was reported to bind directly to TLR2 and initiate downstream signaling through the MyD88/TRAF6/c-Src/p47^phox^ complex [25]. We next investigated whether zymosan activates c-Src and downstream signaling events *via* TLR2 in neutrophils. As a result, knockdown of TLR2, but not TLR4, abolished zymosan-induced expression of iNOS, which is in line with the potent inhibition of NO and ONOO^−^ production by TLR2-targeted siRNAs in zymosan-treated neutrophils (Figures 5A and 5B). Consistently, zymosan induced the activation of c-Src, which is impaired by knockdown of the adaptor protein, MyD88 (Figure 5C). The interaction of c-Src with MyD88, TRAF6 and p47^phox^ upon stimulation with zymosan was detected in neutrophils (Figure 5D). Therefore, TLR2 is responsible for zymosan-induced c-Src activation and downstream signaling leading to NO and ONOO^−^ production in neutrophils.

### Isoflurane impairs c-Src activity by targeting the NMDA glutamate receptor

**Figure 6:**
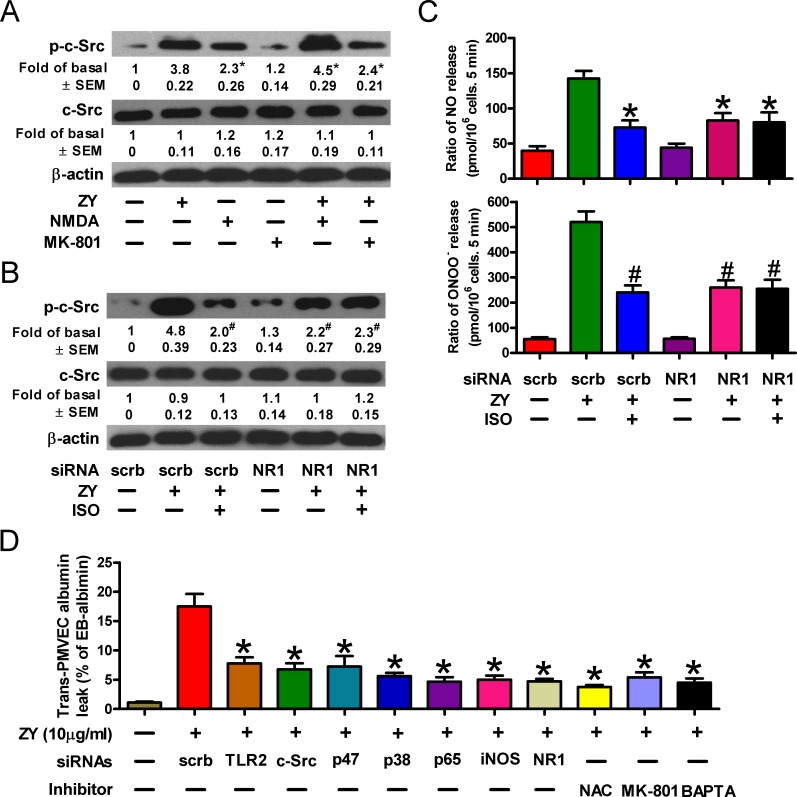
Isoflurane decreases c-Src activity by targeting the NMDA glutamate receptor **A.** Neutrophils were treated with zymosan (10 μg/ml) for 6 h in the presense of the NMDA receptor agonist NMDA (50 mM) or antagonist MK-801 (30 mM) where indicated. Cell lysates were then subjected to Western blot analysis. **B.**, **C.** Neutrophils were transfected with indicated siRNAs prior to treatment with or without zymosan (10 μg/ml). Cells were then subjected to Western blot analysis 6 h later (B) and measurement of NO and ONOO^−^ production 12 h later (C). **D.** Neutrophils were pretransfected with indicated siRNAs, and/or treated with zymosan (10 μg/ml) for 12 h in the presence of the indicated reagents including NAC (50 mM), MK-801 (30 mM) and BAPTA (25 mM). The media were replaced with fresh complete media, which were collected 6 h later and added to the monolayer PMVEC. Trans-PMVEC EB-albumin leak was determined. Data are represented as the mean ± SEM of 3 replicates or representative of 3 independent experiments. *P < 0.05, as compared with the cells exposed to zymosan alone (A). *P < 0.05, ^#^*P* < 0.01, as compared with the cells exposed to zymosan + scrambled siRNA (B-D).

We next deciphered how isoflurane exerts an inhibitory role in c-Src activation and zymosan-primed neutrophilic responses. Isoflurane exerts an anesthetic role by suppressing the receptors of neurotransmitters [12, 26]. The N-methyl-D-aspartic acid (NMDA) glutamate receptors, which can be induced by zymosan in neutrophils, have recently been defined to participate in ALI [27, 28]. We thus evaluated whether NMDA receptors play a role in isoflurane inhibition of zymosan-induced neutrophil signaling. As shown in Figure 6A, NMDA, which is an agonist of the NMDA receptor, recapitulated zymosan-induced activation of c-Src, while the specific NMDA receptor antagonist, MK-801, interfered with c-Src activation in zymosan-treated neutrophils. Knockdown of the NMDA receptor subunit NR1 reduced the levels of phosphorylated c-Src (Figure 6B). Meanwhile, NR1 silencing diminished the suppressive effect of isoflurane on c-Src activation (Figure 6B). In line with these findings, we observed decreased NO and ONOO^−^ production in zymosan-stimulated neutrophils, while further treatment of cells with isoflurane failed to significantly reduce the synthesis of peroxynitrite (Figure 6C). Similar to the media harvested from zymosan-treated neutrophils in the presence of siRNAs or inhibitors targeting the aforementioned TLR2/c-Src/p47^phox^/ROS/p38 MAPK/NF-κB/iNOS pathway, incubation of zymosan-treated neutrophils with MK-801 or the Ca^2+^ chelator, 1,2-bis(2-aminophenoxy)ethane-N, N, N', N'-tetraacetic acid (BAPTA), generated conditioned media with remarkably inhibited capacity to induce trans-PMVEC albumin leak (Figure 6D). These data suggest that NMDA receptors promote zymosan-induced NO and ONOO^−^ production by facilitating c-Src activation, and isoflurane negatively regulates c-Src activity dependent upon NMDA receptor- and calcium-mediated signaling.

### Isoflurane attenuates pulmonary endothelial leak mediated by activated iNOS in zymosan-primed neutrophils in mice

**Figure 7:**
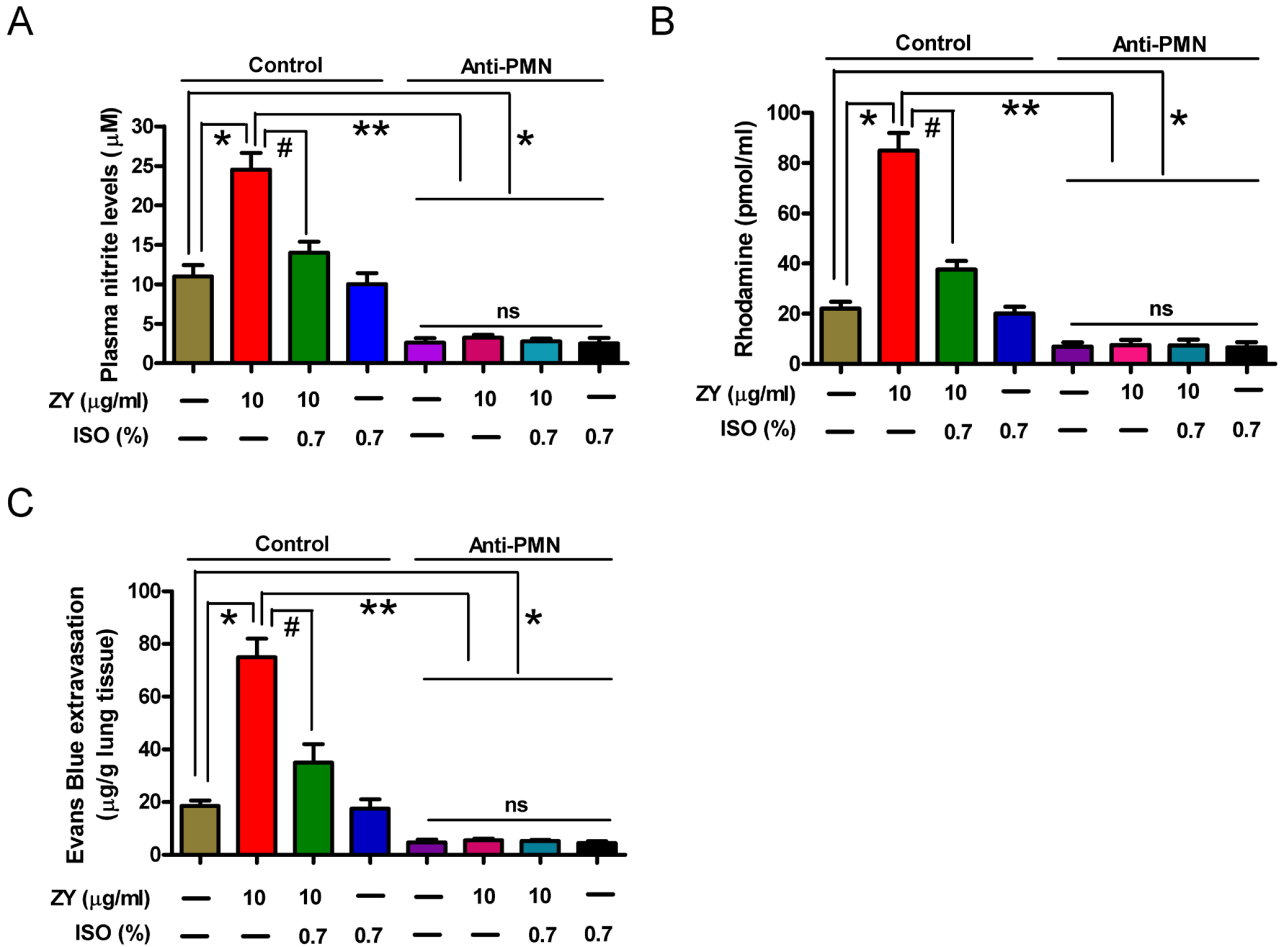
Treatment with anti-PMN antibody reduces zymosan-enhanced plasma NO/ONOO^−^ levels and pulmonary microvascular leak in mice Animals were treated with normal rabbit serum (Control) or anti-PMN antibody as described in the ‘Establishment and assessment of neutrophil depletion in mice’ method section. And the control or anti-PMN (PMN depletion) mice were treated as described in the ‘*In vivo* experimental design’ method section. At 24 h after zymosan or NS administration, plasma nitrite **A.** and peroxynitrite **B.**, and evans blue extravasation in lung **C**. were measured. Data are represented as the mean ± SEM of 3 replicates or representative of 3 independent experiments. *P < 0.05, ^#^*P* < 0.01. ns = Not significantly.

To investigate whether the reduction of lung microvascular injury by isoflurane is dependent on iNOS activation in zymosan-challenged neutrophils in mice, we established a neutrophil-depleted mouse model by intraperitoneal (i.p.) injection of anti-polymorphonuclear leukocyte (PMN) antibody. As shown in Supplementary Figure S2A and S2B, treatment with anti-PMN antibody significantly reduced the percentage of circulating neutrophils and lung MPO activity compared to the control group, suggesting that a neutrophil-depleted mouse model was successfully established. Moreover, we found that zymosan-increased plasma nitrite was dramatically reduced by isoflurane in the control group compared to the anti-PMN group (Figure 7A). Rhodamine measurement experiment showed that isoflurane could markedly impair zymosan-induced formation of peroxynitrite in plasma from the control group compared to the anti-PMN group (Figure 7B). Evans blue extravasation assay demonstrated that isoflurane significantly inhibited zymosan-induced pulmonary microvascular dysfunction in the control group compared to the anti-PMN group (Figure 7C). In addition, zymosan or isoflurane alone or their combination had no effect on plasma levels of NO and ONOO^−^ and pulmonary microvascular leak (Figure 7A-7C). These results indicated that pulmonary microvascular leak under zymosan stimulation conditions is dependent on iNOS activity (NO and ONOO^−^ production) specifically in neutrophils, but not in PMVECs themselves *in vivo*, which was attenuated by isoflurane.

## DISCUSSION

**Figure 8:**
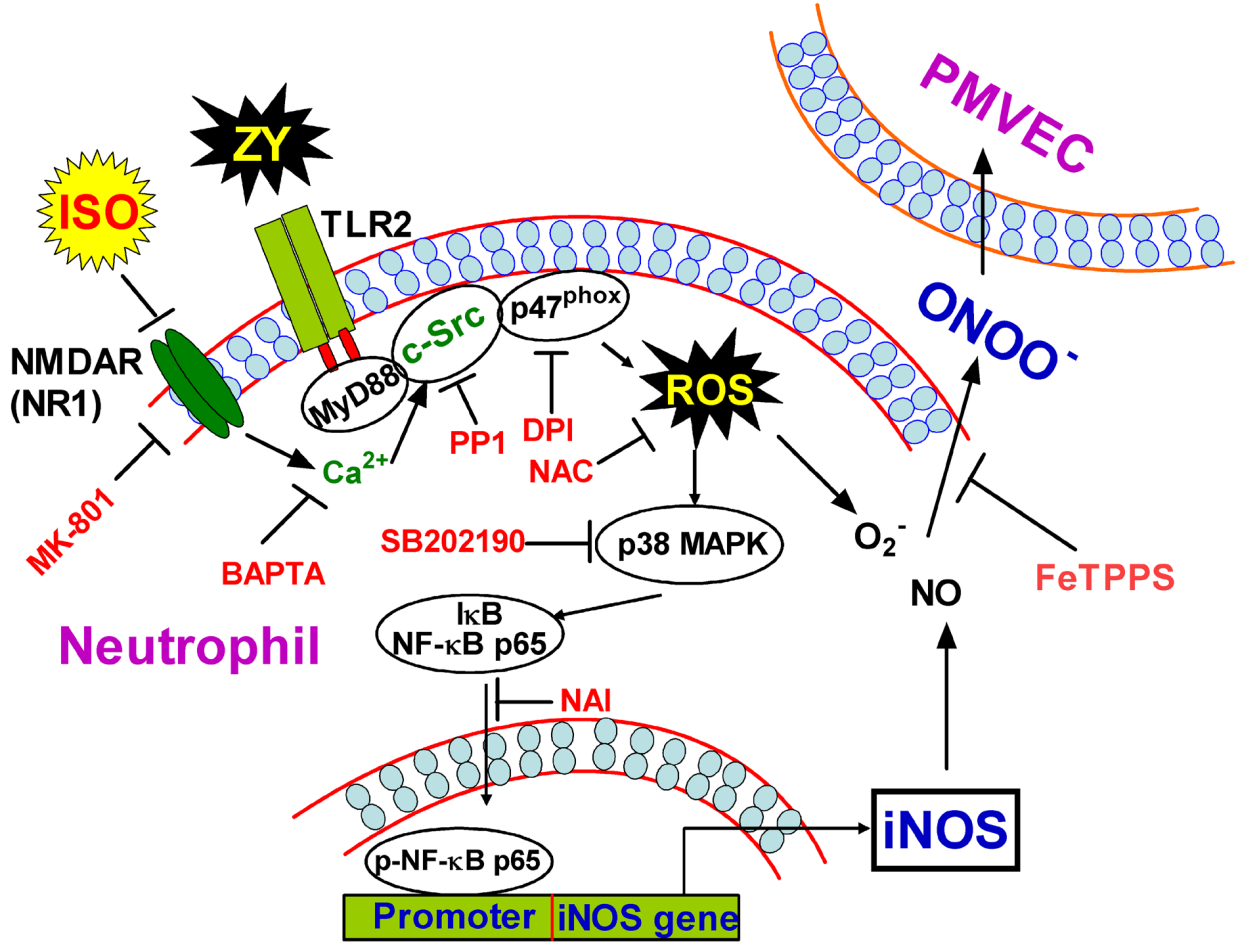
Schematic diagram of the proposed signaling pathways elicited by zymosan but inhibited by subanesthetic isoflurane Zymosan induces ROS production through a TLR2/MyD88/c-Src/NADPH oxidase pathway, which in turn caused the activation of p38 MAPK and consequently the activation and nuclear translocation of NF-κB. NF-κB thus switches on iNOS expression, and increases NO production and ONOO^−^ release from neutrophils, which eventually permeabilizes adjacent PMVECs. Subanesthetic isoflurane exerts a protective role for this pathological process by inhibiting c-Src activity, which is probably attributed to isoflurane targeting of the NMDA glutamate receptor and thereby suppression of the calcium signaling in neutrophils.

Zymosan has been established to induce pulmonary inflammatory responses, thereby providing a canonical model for ALI [5, 6]. Neutrophils play vital roles in zymosan-induced PMVEC damage as exemplified by increased endothelial cell permeability upon neutrophil release of NO and ONOO^−^ [29]. Consistent with previous reports, we found that zymosan elicits neutrophil NO and ONOO^−^ production by promoting iNOS expression, whereas subanesthetic isoflurane plays a protective role in endothelial cell damage by inhibiting this process [7, 30]. We thus deciphered the molecular pathway(s) underlying iNOS expression, and identified that TLR2 signaling and NF-kB are responsible for iNOS expression, which is in agreement with previous findings in NF-kB deficient mice [31]. Toll-like receptors directly activate NF-κB in a canonical pathway *via* a complex involving MyD88, TRAF6 and TGF-β-activated kinase 1 (TAK1), leading to activation of IκB kinase (IKK), degradation of IκB, and nuclear translocation of NF-κB [32]; TLR2 also activates NF-κB through Rac1-PI3K-Akt pathway that does not involve IκB degradation [32]. Our study unraveled an alternative pathway through which TLR2 signaling eventually activates NF-κB in zymosan-primed neutrophils. In this pathway, TLR2 recruits and activates c-Src through MyD88, and c-Src activates NADPH oxidase to generate ROS. ROS subsequently activates p38 MAPK, which triggers nuclear translocation of the p65 subunit of NF-κB to switch on iNOS expression (Figure 8). ALI results from the dysfunction of various types of pulmonary cells, involves extensive crosstalk between inflammatory cells and epithelial cells *via* small molecule mediators or inflammatory cytokines, and is attributed to aberrant activation of diverse receptor pathways such as the documented TLR4 pathway [33, 34]. Nevertheless, our findings revealed the integrated signaling events in the neutrophils that contribute to pathogen-induced inflammation and damage of the pulmonary endothelial cells.

ROS play a key role in the pulmonary vascular abnormalities that characterize ARDS by mediating many forms of neutrophil-dependent endothelial injury in both *in vivo* and *in vitro* models [35, 36]. We established *via* multiple approaches that ROS production is required for zymosan-induced NF-κB activation and iNOS expression in neutrophils, thus excluding the possibility that NF-κB is activated through the canonical TLR/IRAK/TRAF6/IKK pathway. NADPH oxidase enzymes are thought to exert pro-inflammatory roles *via* ROS induction [36]. Consistent with these findings, we showed that zymosan-induced iNOS/NO/ONOO^−^ generation in neutrophils requires NADPH oxidase-mediated ROS production. Zymosan activates NADPH oxidase by inducing membrane translocation of its cytosolic subunit, p47^phox^. In its downstream signaling, cellular ROS activates p38 MAPK, which in turn allows the nuclear translocation of NF-κB probably through the documented IKK/IκB pathway [33]. While ROS and oxidative stress contribute to lung injury in many aspects, and various members of the MAPK family are involved in iNOS regulation, the ROS/p38 MAPK/NF-κB pathway may represent an essential signaling mechanism that mediates the pro-inflammatory role of neutrophils in ALI, e.g. release of nitroxides to permeabilize microvascular endothelial cells in the lung [37, 38]. It is well documented that ROS is critically involved in zymosan-induced ALI [37]. However, the machinery determining ROS production may vary dependent on the cell types in the lung [37, 39]. We established here that c-Src connects TLR2 signaling to NADPH oxidase, leading to ROS production and eventually iNOS expression in zymosan-activated neutrophils, which is in accordance with previous reports that c-Src promotes iNOS expression [40]. To our knowledge, this might be the first characterization of the TLR2/MyD88/c-Src/p47^phox^ complex formation in zymosan-induced NADPH oxidase activation and ROS production. Given the previous finding that c-Src induction by lipopolysaccharide requires NO signaling in the context of macrophage migration, it deserves further investigation whether c-Src and iNOS forms a positive feedback mechanism in zymosan-activated neutrophils [41].

We and others have found that the anesthetic isoflurane exerts a protective role in zymosan-induced inflammation and vascular endothelial cell damage in the lung [7, 30, 42]. In addition to our previous observation that isoflurane counteracts zymosan in regulating iNOS expression in neutrophils [7], we established here that subanesthetic isoflurane relieves zymosan-induced neutrophilic peroxynitrite release and trans-PMVEC protein leak by suppressing the repertoire of c-Src downstream signaling responsible for iNOS expression and peroxynitrite production. More importantly, we found that isoflurane impairs c-Src activity by targeting the NMDA receptor, the subtype of glutamate receptors known to be expressed in neutrophils, thymocytes and lymphocytes [43]. These findings are consistent with recent reports that NMDA receptor antagonists attenuate ALI in various *in vivo* models [27, 28], and in agreement with a previous observation that NMDA-induced retinal neuro/vascular injury, which could be alleviated by the peroxynitrite decomposition catalyst FeTPPs [44]. These data established that isoflurane impedes c-Src activation *via* interfering with NMDA receptor-evoked calcium influx and subsequently calcium-dependent c-Src activation [45-47], suggesting the involvement of NMDA receptor signaling in the maintenance of zymosan-elicited c-Src activity in neutrophils. The regulatory roles of NMDA receptors on c-Src activity and the pro-inflammatory responses of neutrophils are also complicated by the possibility that c-Src may be responsible for a feedback regulation of both NMDA receptor and calcium signaling [48, 49]. Nevertheless, our findings highlight a novel crosstalk between NMDA receptor and Toll-like receptor signal pathways which underlies the protective roles of isoflurane in ALI induced by different pathogens. Moreover, the current study has several limitations. First, we didn't establish neutrophil-PMVEC co-cultures based on a certain ratio for mimicking an *in vivo* environment. Second, this study can be extended to investigate the effect of iNOS in neutrophils on trans-PMVEC protein leak, using the inhibitors of iNOS activity, such as 1400W and L-NAME. Finally, it is unclear whether zymosan-induced pulmonary inflammation and protein leak could be significantly abrogated in iNOS^−/ −^ mice or following iNOS-selective inhibition in iNOS^+/+^ mice. Taken together, the present study unraveled the novel mechanisms of zymosan-induced neutrophil activation and microvascular endothelial dysfunction, and provides the rationale for the subanesthetic isoflurane treatment to protect against lung inflammation and ALI.

## MATERIALS AND METHODS

### Reagents

The antibodies against iNOS, TLR2, TLR4, MyD88, c-Src, p47^phox^, TRAF6, p38 MAPK, Gas, PMN, β-actin, and lamin B were purchased from Abcam (Cambridge, UK). Anti-phospho-c-Src (p-c-Src), anti-phospho-p38 MAPK (p-p38 MAPK), and anti-phospho-NF-κB p65 (p-NF-κB p65) antibodies were obtained from Cell Signaling Technology (Danver, MA, USA). Diphenyleneiodonium chloride (DPI), N-acetylcysteine (NAC), PP1, and SB202190 were acquired from Biomol (Plymouth Meeting, PA, USA). NAI and BAPTA were obtained from Calbiochem (Darmstadt, Germany). 2ʹ, 7ʹ-dichlorodihydro-fluorescein diacetate acetyl ester (DCFDA) was purchased from Molecular Probes (Eugene, OR, USA). Isoflurane was purchased from Baxter (Baxter Healthcare Corporation, Deerfield, USA). Zymosan, FeTPPS, 3-morpholinosydnonimine (SIN-1), NMDA and MK-801 were obtained from Sigma (St. Louis, MO, USA). Zymosan was dissolved in normal isotonic sodium chloride solution (NS) to a final concentration of 25 mg/mL. The solution was homogenized by magnetic stirring, and sterilized at 100°C for 80 min. All suspensions were freshly prepared prior to use.

### Neutrophil isolation, culture and treatment

Neutrophils were isolated from peripheral venous blood of mice using an anti-Ly-6G MicroBead Kit (MiltenyiBiotec, Germany) according to the manufacturer's protocol. Prior to all experiments, > 99% of cells were determined viable by Live/Dead violet (Invitrogen, Carlsbad, CA). Neutrophils were seeded on 6-well plates, allowed to incubate overnight, and then subjected to various experimental conditions at 37°C. After reducing the media volume in each well from 2.5 mL to 1 mL, cells were exposed to room air (RA) with or without isoflurane at 2 L/min in a metabolic chamber (Columbus Instruments, Columbus, OH) after various treatments. During isoflurane exposure, the isoflurane concentration (0.7%) was continuously verified by sampling exhaust gas with a DatexCapnomac (SOMA Technology Inc., Cheshire, CT).

### Transit transfection with siRNAs

The small interfering RNA (siRNA) duplexes corresponding to mouse TLR2 (sc-40257), TLR4 (sc-40261), MyD88 (sc-35987), c-Src (sc-29859), p47^phox^ (sc-151963), p38 MAPK (sc-29434), NF-κB p65 (sc-29411), iNOS (sc-36092), NMDA receptor subunit NR1 (sc-36082), and scrambled siRNA (sc-44230) were purchased from Santa Cruz Biotechnology, Inc. (Dallas, Texas, USA). SiRNA transfection was carried out using lipofectamine^®^ 2000 transfection reagent from Invitrogen (Carlsbad, CA, USA). SiRNAs (100 nM) were formulated with Lipofectamine^®^ 2000 transfection reagent according to the manufacturer's instructions.

### PMVEC isolation, culture and treatment

Mouse PMVECs were isolated by digesting the whole lung tissue with 0.5 mg/mL collagenase IA (Sigma) for 45 min at 37°C. The digested suspension was filtered through 70 mm mesh, centrifuged at 250 *g* for 4 min, and washed twice in phosphate-buffered saline (PBS). The cell suspension was incubated with magnetic microbeads (Dynal Inc., Lake Success, NY) coated with rat anti-mouse CD31 antibody (BD Pharmingen, USA) for 20 min at 4°C to select out PMVEC for culture. To ensure culture purity, cultures were repurified with anti-CD31-coated magnetic microbeads, as described above, after the first passage. Dil-conjugated Ac-LDL (Dil-Ac-LDL) and CD31 staining showed more than 95% purity for PMVECs. PMVECs were used for experiments at passages 3 to 4. To investigate the effect of zymosan on PMVEC, 10 μg/ml zymosan was added into PMVEC cultures.

### Trans-PMVEC albumin leak

Conditioning media from neutrophils treated with siRNAs, inhibitors or 0.7% isoflurane were added to the PMVEC monolayer seeded in 10% gelatin coated Transwell inserts for 1 h. After EB-conjugated albumin was added to the upper chamber for 1 h, the media in the lower chamber were collected, and the absorbance of EB was measured at 595 nm and referred to a standard curve in order to report trans-PMVEC EB-albumin leak as % of total EB-albumin in the upper compartment into the lower compartment.

### RT-PCR analysis

Total RNA of neutrophils subjected to various treatments was extracted using TRIzol^®^ Reagent (Invitrogen, Carlsbad, CA, USA) following the instructions of the manufacturer. The cDNA obtained from 1 mg of total RNA was used as a template for PCR amplification. The primers were the following sequences: iNOS, forward 5ʹ-TGAACCCCAAGAGTTTGACC-3ʹ and reverse 5ʹ-TGCTGAAACATTTCCTGTG C-3ʹ; β-actin, forward 5ʹ-GTGGGCCGCTCTAGGCACCA-3ʹ and reverse 5ʹ-TGGCC TTAGGGTGCAGGGGG-3ʹ. DNA band densities were quantified using Quantity One software (BioRad, USA). The RT-PCR data were normalized with the internal control β-actin and then calculated as fold changes compared with the first treatment group (fold of basal is 1 and ± SEM is 0) in each experiment.

### Isolation of cell fractions

Neutrophils were harvested, sonicated for 6 s at output 1.5 with a sonicator (Misonix Inc., Farmingdale, NY), and then centrifuged at 8,000 rpm for 15 min at 4°C. The pellet was collected as the nuclear fraction. The supernatant was centrifuged at 14,000 rpm at 4°C for 60 min to yield the supernatant (cytosolic fraction) and the pellet (membrane fraction).

### Western blot analysis

The whole lysates or cytosolic or nuclear extracts of neutrophils or PMVECs were prepared, separated by sodium dodecyl sulfate polyacrylamide gel electrophoresis (SDS-PAGE), electrotransferred onto nitrocellulose membranes, and then immunoblotted with primary antibodies. Equivalent sample loading was confirmed by probing the levels of β-actin (for whole lysates or cytosolic proteins), lamin B (for nuclear proteins), and Gas (for membrane proteins). Detection was performed with the enhanced chemiluminescence assay kit (Pierce, Rockford, IL, USA). Protein band densities were quantified using Quantity One software (BioRad, USA). The Western blot results were normalized with the internal control β-actin or lamin B and then calculated as fold changes compared with the first treatment group (fold of basal is 1 and ± SEM is 0) in each experiment.

### Co-immunoprecipitation assay

Neutrophil lysates containing 2 mg of protein were incubated with 5 mg anti-c-Src antibody at 4°C for 24 h. Then, 20 mL of 50% protein A-agarose beads was added and gently mixed at 4°C for 24 h. The immunoprecipitates were collected and washed thrice using a lysis buffer without Triton X-100. A 5 × Laemmli buffer was added; the mixture was subjected to electrophoresis on SDS-PAGE, and then blotted with anti-TLR2, anti-MyD88, anti-TRAF6, anti-c-Src, and anti-p47^phox^ antibodies.

### iNOS promoter activity assay

The pGL-iNOS promoter-Luciferase reporter plasmid was purchased from Addgene (Cambridge, MA, USA), and was used for transfection of neutrophils. iNOS-luc activity was determined using a luciferase assay system (Promega, Madison, WI, USA).

### Measurement of neutrophil NO production

At the indicated time points, neutrophils subjected to various treatments were harvested. The chemical determination of NO is based on the diazotization of sulphanilamide by NO at acidic pH and the subsequent oxidation of scopoletin, which can be fluorophotometrically detected as previously described [50].

### Analysis of neutrophil ONOO^−^ release

ONOO^−^ was measured by luminol-amplified chemiluminescence. All light (photons) emitted was measured using a Berthold AutoLumat LB953 Luminometer (Dr Berthold GmbH & Co. KG, Wildbad, Germany). Neutrophils subjected to various treatments were placed in a vial. Light emission was recorded by a computer interface and reported as the integrated light emission for a total period of 0.05-1.00 s. The results were calculated in counts per second. Chemiluminescence responses were converted to picomoles of ONOO^−^ using a standard curve constructed with various concentrations of pure ONOO^−^.

### Measurement of ROS production

The intracellular H_2_O_2_ levels were determined by measuring fluorescence of DCFH-DA. Neutrophils were washed with warm HBSS and incubated in HBSS containing 10 mM DCFH-DA at 37°C for 30 min, and then replaced with fresh media. Cells were washed twice with PBS, and the fluorescence was captured with confocal laser scanning microscopy (Nikon, Tokyo, Japan). Alternatively, the fluorescence intensity of the cells was analyzed using a FACScan flow cytometer (BD Biosciences, San Jose, CA) at 495 nm excitation and 529 nm emission.

### Determination of NADPH oxidase activity

A lucigenin chemiluminescence assay was used to measure NADPH oxidase activity in cells as previously described [51]. Briefly, neutrophils were seeded in 24-well plates, subjected to various treatments at the indicated times, gently harvested, and centrifuged at 1,200 rpm for 15 min at 4°C. The cell pellet was resuspended with 50 mL/per well of ice-cold RPMI 1640 medium. A 5 mL portion of the cell suspension (0.2 × 10^5^ cells) was added to a final 180 mL of pre-warmed (37°C) RPMI 1640 medium containing either lucigenin (20 mM) or NADPH (1 mM) to initiate the reaction, followed by immediate measurement of chemiluminescence using the Appliskan luminometer (Thermo^®^) in an out-of-coincidence mode. Appropriate blanks and controls were established, and chemiluminescence was recorded continuously for 15 min. The activity of NADPH oxidase was expressed as counts per million cells.

### Establishment and assessment of neutrophil depletion in mice

Mice were administered with an i.p. injection of either normal rabbit serum as control or rabbit polyclonal antibody to mouse neutrophils (anti-PMN; AIAD51140; Accurate Chemical Co, Westbury NY). The initial dose was given at 1 mL/g body weight. Additional i.p. injections of control or anti-PMN were performed at 1.5 mL/g body weight on 2nd, 4th, and 5th day. Sequential saphenous vein bleeds were used to assess the effectiveness and specificity of the antisera in neutrophil depletion.

Blood was collected in EDTA coated tubes and total white blood cells and platelets were counted by standard methods in a hemacytometer. Hematocrit was determined on 5th day post initial injection with blood obtained from the carotid and aorta, respectively. Differential counts were obtained from blood smears stained with a modified Wrights' stain (Diff Quik, American Scientific Products, McGraw Park, IL). Two hundred cells were counted and identified as eosinophils, neutrophils, lymphocytes or monocytes. Monocytes and eosinophils typically comprised less than 1% of the cell differential in all of the treatment groups and so they were not included in the analysis.

For estimates of the numbers of neutrophils in tissue, lung myeloperoxidase (MPO) activity was assessed as previously described [52]. On 5th day post initial injection of control or anti-PMN, all animals (n = 10 for each group) were sacrificed with sodium pentobarbital. Lungs were obtained and perfused with cold PBS to remove all blood, and homogenerated lung supernatants were prepared for detecting MPO activity, which was defined by spectrophotometer (DU 640B; Beckman) at 590 nm using commercial kits and expressed in unit per gram weight of wet tissue.

### Zymosan-induced lung injury in mice and isoflurane treatment

A zymosan-induced lung injury model was established by asepticly i.p. injection of zymosan into mice at a dose of 1 g/kg of body weight, as previously described [30]. The same volume of NS was injected through the same route to serve as the sham control. To verify the anti-inflammatory role of 0.7% isoflurane, the mice (Control or anti-PMN) were placed in a sealed Plexiglass chamber with inflow and outflow outlets and isoflurane was delivered by air flow into the chamber through a tube at a rate of 4 L/min. The flow rate of isoflurane was accurately controlled in real-time by regulation of Anesthetic Vaporizers (Harvard apparatus, USA). Isoflurane concentration in the outflow hose of the chamber was continuously monitored with a gas analyzer (Brϋel & Kjaer, Naerum, Denmark) and maintained at 0.7% during the treatment. Oxygen concentration in the chamber was maintained at 21% using supplemental oxygen and continuously monitored with a gas analyzer (Medical Gas Analyzer LB-2, Model 40 M; Beckman, Fullerton, CA, USA). Carbon dioxide was removed from the chamber gases with Baralyme (Allied Healthcare Products, Inc., St. Louis, MO, USA). Animals without isoflurane treatment were exposed to RA in the chamber as the vehicle control. The room and chamber temperatures were maintained within 22-24°C.

### *In vivo* experimental design

Eighty mice (Control) and another Eighty mice (anti-PMN) were randomly allocated as follows, respectively (each group = 20): (1) Zymosan (ZY) + vehicle group: control or anti-PMN mice were given an i.p. injection of ZY, followed by inhalation of RA (vehicle) for 1 h starting at 1 h and 6 h after ZY administration. (2) ZY + isoflurane (ISO) group: no differences from the ZY + vehicle group, except for 1 h inhalation of 0.7% ISO starting at 1 h and 6 h instead of RA after ZY administration. (3) Sham + vehicle group: no differences from the ZY + vehicle group, except for administration with NS (Sham) instead of ZY. (4) Sham + ISO group: identical to the sham + vehicle group, except for 1 h inhalation of 0.7% ISO starting at 1 h and 6 h instead of RA after NS (sham) administration. At 24 h after ZY or NS administration, the below assays were carried out.

### Measurement of plasma nitrite

Production of nitrite (NO_2_^−^), an indicator of NO synthesis, was assessed using a colorimetric reaction with the Griess reagent [53]. Plasma was collected and mixed with an equal (1:1) volume of Griess reagent [0.1% N-(1-naphthyl) ethylenediamine dihydrochloride, 1% sulfanilamine, and 2.5% H_3_PO_4_]. A 96-well microplate reader (Spectra MAX 340PC, Molecular Devices) was used to measure the absorbance at 540 nm. Data were analyzed using Softmax Pro software. Sodium nitrite was dissolved in double-distilled water for use as standards.

### Measurement of DHR123 oxidation in plasma

The ONOO^−^-dependent oxidation of DHR123 to rhodamine was used to determine the formation of ONOO^−^ in plasma, as described by Kooy et al. [54]. Animals were intravenously administered with DHR123 (2 mM/kg in 0.05 mL of saline) and sacrificed 15 min after injection. A fluorometer was used to measure rhodamine fluorescence for plasma samples with an excitation wavelength of 500 nm and emission wavelength of 536 nm. The rate of rhodamine formation, an index of ONOO^−^ production, was calculated against a standard curve obtained with authentic rhodamine prepared in plasma from untreated mice.

### Evans blue extravasation assay

ZY-induced pulmonary microvascular dysfunction was quantified by measuring the concentration of EB dye within the lung after intravenous injection of dye. EB dye binds avidly to albumin as a marker of protein extravasation in models of inflammatory tissue injury [16]. At the indicated time points, mice were anesthetized by i.p. injection of sodium pentobarbital, and EB dye (30 mg/kg) was slowly injected to the right femoral vein and the bilateral lungs were harvested 30 min after infusion. The pulmonary vasculature was cleared of blood, and the lungs were weighed and placed in 1 ml of formamide at 37°C overnight. The absorbance of EB dye in eluate was photometrically measured at 650 nm (Zeiss DMR 10, Germany). Extravasated EB extracted from the lungs was calculated against a standard curve and expressed as micrograms of dye per gram of lung tissue (mg/g tissue).

### Statistical analysis

Quantitative data were expressed as means ± SEM. Groups were compared using Student's two-tailed unpaired *t* test or one-way ANOVA analysis, followed by Dunnet's post-hoc test as appropriate. *P* < 0.05 was considered significant. Data were estimated using a GraphPad Prism Program (GraphPad, San Diego, CA).

## SUPPLEMENTARY FIGURES



## Abbreviations

ALI: acute lung injury
ARDS: acute respiratory distress syndrome
iNOS: inducible NO synthase
TLR2: toll-like receptor 2
NMDA: N-methyl-D-aspartic acid
PMVEC: pulmonary microvascular endothelial cell
NADPH: nicotinamide adenine dinucleotidephosphate
IKK: IκB kinase
