# Supplementary material for: Subanesthetic isoflurane relieves zymosan-induced neutrophil inflammatory response by targeting NMDA glutamate receptor and Toll-like receptor 2 signaling
Source: Oncotarget. 2016 Apr 28;7(22):31772–89. doi: 10.18632/oncotarget.9091 (PMC5077975; doi:10.18632/oncotarget.9091)
Supplement: Supplementary file 1 [file oncotarget-07-31772-s001.pdf]

Subanesthetic isoflurane relieves zymosan-induced neutrophil inflammatory response by targeting NMDA glutamate receptor and Toll-like receptor 2 signaling

Supplementary Material

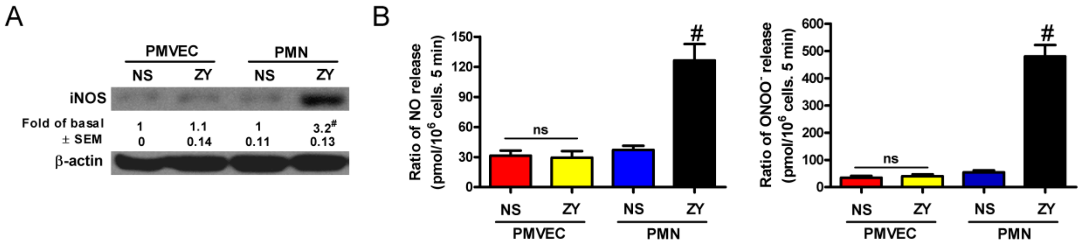

Figure S1: Zymosan induced iNOS expression and activation in PMN but not in PMVEC. (A) PMN or PMVEC was treated with NS or zymosan for 8 h before Western blot analysis. β-actin was used as an inner control for whole cell lysates. (B) PMN or PMVEC was treated with NS or zymosan for 12 h, followed by measurement of NO and $ONOO^-$ production. Data are represented as the mean ± SEM of 3 replicates. $^{\#}P < 0.01$, as compared with PMVECs exposed to NS or zymosan (A-C), or PMN exposed to NS. ns = Not significantly.

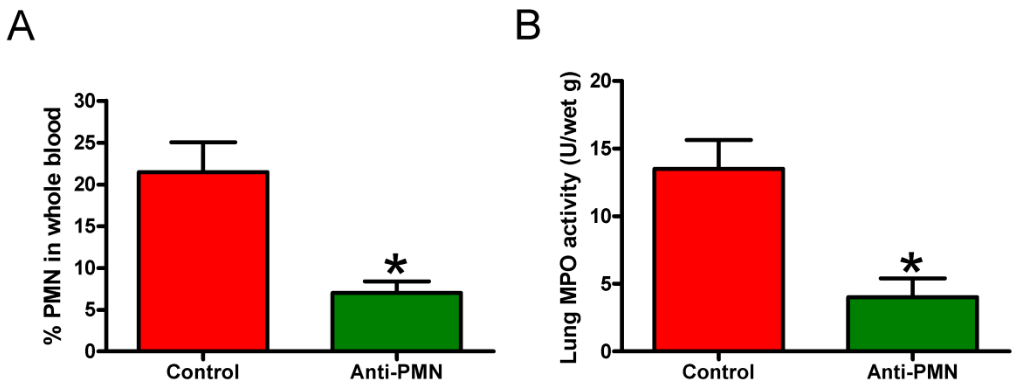

Figure S2: Treatment with anti-PMN antibody reduces the percentage of circulating neutrophils and lung MPO activity. Animals were treated with normal rabbit serum (Control) or anti-PMN antibody as described in the 'Establishment and assessment of neutrophil depletion in mice' method section. (A) Anti-PMN treatment significantly reduced circulating neutrophils compared to normal rabbit serum treatment. (B) Lung MPO activity was used as an indicator of the number of neutrophils in the lung. Anti-PMN treatment significantly reduced MPO activity compared to normal rabbit serum treatment. Data are represented as the mean ± SEM of 3 replicates or representative of 3 independent experiments. *P < 0.05, as compared with the control groups.
